# Editorial: New strategies to overcome platinum resistance in ovarian cancer

**DOI:** 10.3389/fonc.2024.1390760

**Published:** 2024-03-14

**Authors:** Jian-Jun Wei, Fabio Martinelli

**Affiliations:** ^1^ Department of Pathology, Northwestern University Feinberg School of Medicine, Chicago, IL, United States; ^2^ Department of Gynecologic Oncology, National Cancer Institute Foundation (IRCCS), Milan, Italy

**Keywords:** ovarian cancer, cancer therapy, carboplatin resistance, editorial, drug development

Ovarian cancer is a lethal disease with a high mortality rate and is the fifth leading cause of cancer death in women. Current front-line treatments use platinum-based anticancer agents, such as cisplatin and carboplatin. However, Ovarian cancer inevitably acquires resistance to these drugs when cancer cells become insensitive to the effects of platinum-based chemotherapy, leading to treatment failure and disease progression (1, 2). Ovarian cancer cells with carboplatin resistance develop new mutations to block carboplatin, enhance DNA repair mechanisms by repairing the damage caused by carboplatin (3), create a local microenvironment with a protective shield blocking carboplatin penetration, develop mechanisms to pump out the chemotherapy drugs, and manipulate the signaling pathways for less responsive to carboplatin treatment (4). Furthermore, ovarian cancer is known for its heterogeneity resulting in tumor cells with varying responses to treatment (5). The major challenges in managing ovarian cancer are the development of new strategies for early identification of the genetic and environmental factors that are associated with chemo-resistance in ovarian cancer cells (6, 7). Thus, promoting medical research to explore the mechanisms underlying carboplatin resistance in ovarian cancer is crucial which will aid in new diagnostic tools and more effective drugs to predict and overcome platinum resistance in ovarian cancer.

In this Research Topic, we aim to promote discussion on the emerging molecular studies and clinical strategies for improving our understanding of carboplatin resistance. We are pleased to present a series of original investigation and review articles from the new and well-established experts in the field. The selected research works on this topic hold their preliminary and promise findings and data with the different approaches for carboplatin resistance. The topics discussed in this series will be of relevance to a wide audience, from basic academic researchers to clinicians in gynecologic oncologists, geneticists, and patients.

Development of drug delivery systems and formulations is one of promising approach to improve the pharmacokinetics and tumor accumulation of carboplatin. Nanoparticle-based delivery systems, liposomal formulations, and targeted drug delivery approaches may enhance drug delivery to tumor sites and overcome resistance mechanisms. The first innovative study by Ashoori et al. reported their study of “*Polyethylenimine-based iron oxide nanoparticles to enhance cisplatin toxicity in ovarian cancer cells in the presence of a static magnetic field*”. Authors developed Polyethylenimine (PEI)-based magnetic iron oxide nanocomplexes for drug delivery in genetically matched CIS-resistant (A2780/CP) and -sensitive (A2780) ovarian cancer cells.

Advancement of precision medicine approaches to tailor treatment strategies based on the individual patient’s tumor characteristics, genetic profile, and time to adjuvant chemotherapy (TTC). Personalized treatment algorithms incorporating genomic data, molecular profiling, and functional assays may optimize treatment selection and improve patient outcomes. Farolfi et al. reported their study of “*Impact of the time interval between primary or interval surgery and adjuvant chemotherapy in ovarian cancer patients*” and they demonstrated that a TTC ≥ 60 days in primary debulking surgery (PDS) was associated with a shorter PFS.

Identification of novel targets and pathways involved in carboplatin resistance is crucial to uncover alterations associated with resistance and develop targeted therapies against these mechanisms. Immunotherapy approaches, including immune checkpoint inhibitors, cancer vaccines, adoptive cell therapies, and other strategies can enhance immune responses against ovarian cancer. Understanding the immune evasion mechanisms in carboplatin-resistant tumors and developing strategies to overcome them will be important. Mairinger et al. performed gene expression analysis for anti-cancer immunogenicity-related targets using the NanoString nCounter platform and identified 770 targets and 30 reference genes. They identified that a high expression of tumor-associated Metallothionein (MT) was significantly associated with prolonged progression-free and overall survival. They demonstrated MT regulated T-cell receptor gene signature and interferon-gamma expression which were the potential targets for anti-cancer immune response in MT-positive ovarian cancer.

The genomic, transcriptomic, and proteomic profiling can facilitate the new discovery of molecular targets and specific pathways for carboplatin resistance and help to develop targeted therapies against these mechanisms. Study by Eskander et al. observed that long term direct co-culture of sensitive and resistant ovarian cancer cells promoted proliferation of sensitive cells through enrichment of cell cycle control and cell cycle regulation pathways, detected by transcriptomic analysis. The transcription factor E2F1 was predicted as the main effector responsible for the proliferation of sensitive cells. They suggested that E2F1 mediated competition, proliferation, and response to cisplatin in cohabitating resistant and sensitive ovarian cancer cells.

Three review articles in this series provide comprehensive and update reports summarizing the recent progresses of carboplatin resistance in ovarian cancer. Li et al. performed a meta-analysis for different combined treatment options for recurrent platinum-resistant ovarian cancer. The data demonstrated a better overall survival in adavosertib + gemcitabine than conventional and other combined regimen. In fact, cancer cells may develop cross-resistance to multiple chemotherapeutic agents, including platinum-based drugs and other classes of cytotoxic agents. Overcoming resistance to one drug often does not guarantee sensitivity to others, necessitating the development of other therapeutic approaches. Apparently, such analysis may open a venue for the novel combination therapies involving carboplatin with other chemotherapy agents, including targeted therapies, immunotherapies, or agents. Rational combinations that target multiple pathways implicated in resistance may enhance treatment efficacy and overcome drug resistance.

Altered metabolic pathway is one of major molecular mechanisms leading to platinum resistance. Yan et al. offered a comprehensive review of the metabolic processes of the three major nutrients, which are interrelated but can influence each other. For example, glucose can be converted into fat, and the intermediate products of sugar metabolism can generate non-essential amino acids. The changes in key enzymes of upstream genes, and downstream targets in the process can be stimulated by cytotoxic drugs and result in a variety of metabolic processes in ovarian cancer cells and their tumor microenvironment. The key metabolic pathways may be used as therapeutic targets to improve platinum-resistant relapse.

The review article provided by Eskander et al. covers the most important issues, questions, and challenges of drug development in platinum-resistant ovarian cancer. This can be demonstrated by illustrating the landscape in platinum-resistant ovarian cancer and providing the history, current progress and future direction of this topic. The new discovery for overcoming carboplatin resistance is desperately needed to save life for patients with ovarian cancer. The future studies of research priorities will be mainly focused on 1) identification of new targets; 2) combined therapies; 3) immunotherapies; 4) biomarker development; 5) drug delivery system; 6) precision medicine approach; 7) preclinical model and drug screening (1, 8–11). Any achievement for above study subjects will unravel the complexity of carboplatin resistance in ovarian cancer and develop more effective strategies to overcome resistance, prolong patient survival, and improve quality of life.

At end of this Research Topic, we would sincerely thank all the authors and research team for the dedication and contribution of their high-quality articles.

## Author contributions

J-JW: Conceptualization, Project administration, Resources, Supervision, Writing – original draft, Writing – review & editing. FM: Conceptualization, Project administration, Supervision, Writing – review & editing.
